# Psychometric Assessment of the Injection Pen Assessment Questionnaire (IPAQ): measuring ease of use and preference with injection pens for human growth hormone

**DOI:** 10.1186/1477-7525-10-126

**Published:** 2012-10-09

**Authors:** Andreas M Pleil, Miriam Kimel, Julie McCormack, Natasa Rajicic, Judith Hey-Hadavi

**Affiliations:** 1 Pfizer, Inc, 10555 Science Center Dr San Diego, CA, 92121, USA; 2 United BioSource Corporation, MD, Bethesda, USA; 3 Oxford Outcomes, MD, Bethesda, USA; 4 Pfizer Inc, New York, USA

**Keywords:** Patient-reported outcome, Injection device (pen), Ease of use, Parent–child dyads, Growth hormone (GH)

## Abstract

**Purpose:**

To examine the psychometric properties of the Injection Pen Assessment Questionnaire (IPAQ) including the following: 1) item and scale characteristics (e.g., frequencies, item distributions, and factor structure), 2) reliability, and 3) validity.

**Methods:**

Focus groups and one-on-one dyad interviews guided the development of the IPAQ. The IPAQ was subsequently tested in 136 parent–child dyads in a Phase 3, 2-month, open-label, multicenter trial for a new Genotropin® disposable pen. Factor analysis was performed to inform the development of a scoring algorithm, and reliability and validity of the IPAQ were evaluated using the data from this two months study. Psychometric analyses were conducted separately for each injection pen.

**Results:**

Confirmatory factor analysis provides evidence supporting a second order factor solution for four subscales and a total IPAQ score. These factor analysis results support the conceptual framework developed from previous qualitative research in patient dyads using the reusable pen. However, the IPAQ subscales did not consistently meet acceptable internal consistency reliability for some group level comparisons. Cronbach’s alphas for the total IPAQ score for both pens were 0.85, exceeding acceptable levels of reliability for group comparisons.

**Conclusions:**

The total IPAQ score is a useful measure for evaluating ease of use and preference for injection pens in clinical trials among patient dyads receiving hGH. The psychometric properties of the individual subscales, mainly the lower internal consistency reliability of some of the subscales and the predictive validity findings, do not support the use of subscale scores alone as a primary endpoint.

## Introduction

Human growth hormone (hGH) is produced and excreted by the anterior pituitary gland to help fuel growth during childhood and to maintain tissues and organs throughout life [1]. In recent decades, recombinant hGH has been used to treat short stature or growth failure in children, including the following: 1) growth hormone deficiency, 2) born small for gestational age, 3) Prader-Willi syndrome, 4) Turner syndrome, 5) chronic renal insufficiency, and 6) idiopathic short stature. Most children receive injections daily and treatment usually is carried out over several years, until the child achieves an acceptable adult height or maximum growth [2].

In order to achieve optimal therapeutic results, adherence to long-term, continuous hGH administration is essential [3]. Ease of use is recognized by parents, physicians and nurses as a key feature in device acceptance [3] with potential to improve adherence. Injection devices, such as pre-filled syringes and manual injector pens, have been developed to make the process of preparing and administering hGH easier and more convenient. There are several different injection devices available on the market, including the Genotropin® pen (i.e., reusable pen). Several steps must be completed in order to prepare the pen and to inject the hGH, including inserting a cartridge, mixing the medicine, inserting a needle, getting rid of air bubbles, using the needle guard, choosing or dialing the dose and finally injecting hGH. Depending on the age of the child, either the parent, the child, or both participate in preparing the injection pen and administering the medication. As such, patient-reported outcomes (PRO) measures developed to assess these injection devices should include feedback from both parents and children to better reflect how these devices are used in practice.

Another reason for obtaining parent–child opinions together is based on findings from the literature suggesting variability in perceptions of disease impact between parent and child in health-related quality of life (HRQL) [4-8]. It has been suggested that good parent–child agreement is seen for items that are concrete and observable (e.g., physical aspects of health), and poor agreement for items that require judgment (e.g., emotional or social aspects of health). However, a recent review of the literature suggests that levels of parent–child agreement may be influenced by the relevance of a domain to a disease and to the consequent parental involvement to care for the child [8], not merely by the objectivity of the domain. That is, parent–child agreement may be higher when the parent is more involved in caring for the child in a domain that is more influenced by disease. For example, in patients with rheumatoid arthritis, in which physical function is impacted, parents may need to provide more assistance with physical activities and may be more aware of their child’s physical functioning, resulting in stronger agreement on domains that measure the physical impact of disease. In this population, concordance in HRQL scores between parents and children was 0.71 for physical functioning, which was stronger than concordance for emotional functioning and worry (r = 0.51 and 0.48, respectively) [9].

For measures that evaluate domains or activities with high parent–child involvement, such as injection pen preparation and use, a dyadic approach may be useful to overcome concordance issues that arise in obtaining information separately from the parent-child. Although little research has been conducted to evaluate the parent-child dyad relationship in developing PRO questionnaires or in responding to PRO questionnaires, a qualitative analysis of parent–child dyad approach by Ungar and colleagues [10] suggests that a dyadic approach could be helpful to children in enabling them to answer questionnaire items as accurately as possible. When responding to HRQL questionnaires together, parents were a valuable resource to their children (ages 8–15 years) and helped them overcome problems with recall or comprehension that they may have had with the questionnaire. It was noted that child participants would look to their parents to corroborate answers, help remember events and clarify the meaning of questions, words or phrases. Additional studies are needed to better understand this methodological approach, including evaluations of the psychometric properties of measures that are administered to parent and children together.

The Injection Pen Assessment Questionnaire (IPAQ) was developed to evaluate patient (i.e., children 8 to 18 years old) and parent perceptions of ease of use and preference for attributes of injection pens used to administer hGH. The questionnaire was designed to be administered to parent–child dyads, where dyads together are asked to complete a single copy of the questionnaires. The objectives of this study were to examine the psychometric properties of the IPAQ including the following: 1) item and scale characteristics (e.g., frequencies, item distributions, and factor structure), 2) reliability, and 3) validity.

## Study methods

### Instrument development

**Figure 1:**
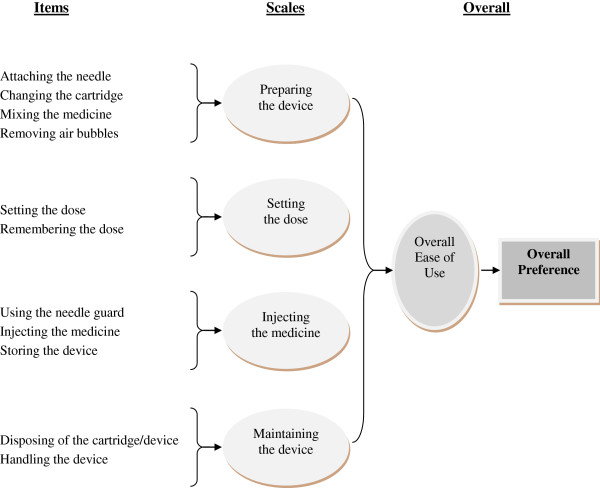
Conceptual Framework Model for the Injection Pen Assessment Questionnaire.

The IPAQ was developed through a scientifically rigorous, systematic process [11]. First, four focus groups with parent–child dyads were conducted to identify key issues and concerns about use of injection pens to administer hGH, as well as to learn the language that dyads use to describe the attributes of injection pens. Based on information collected, a working draft questionnaire was developed. Following this, one-on-one cognitive debriefing interviews were conducted with eight parent–child dyads with previous experience using a pre-specified injection pen to ensure that items in the working draft questionnaire were easy to complete, well understood and relevant to their experience. Findings obtained from each step in the item generation and selection process were reviewed by the research team, including two psychometricians and two clinicians (one endocrinologist), >and provided recommendations to ensure face validity. In addition, a translation expert reviewed the draft questionnaire and provided feedback about words or phrases that may be structurally or culturally problematic when translated into different languages. A total of 29-items were included in the draft IPAQ: 1) fourteen items evaluating ease of use for a single injection pen, 2) fourteen items comparing ease of use between injection pens, and 3) one item evaluating preference for an injection pen. The conceptual framework was established (Figure 1) based on the results of the focus group and cognitive debrief activities. Four components of ‘ease of use” were hypothesized; 1) preparing the pen; 2) setting the dose; 3) injecting the medicine and; 4) maintaining. The questionnaire was then administered to 136 dyads experienced in using hGH injections in a prospective study with a secondary objective to evaluate its psychometric characteristics (i.e., reliability, validity). The results of the study’s primary endpoint are reported elsewhere [12].

### Psychometric evaluation study

One-hundred-thirty six parent–child dyads were recruited from 20 study sites across the United States into a Phase 3 trial to evaluate a new Genotropin® disposable injection pen (“new disposable pen”). The trial was an open-label, non-comparative, single-arm, multi-center study with a duration of treatment of 2 months. All subjects, based on the child member of the dyad, met the following inclusion criteria: 1) age ≥8 years and ≤18 years, 2) currently on treatment with Genotropin® Pen ≥3 months, 3) compliant with current Genotropin® Pen treatment, 4) able to understand English, and 5) have a negative urine pregnancy test at screening, if of childbearing potential. If possible, assent was obtained from the child. Adult members of the dyad needed to: 1) provide written informed consent and 2) be able to read English and understand English. In addition, dyads needed to be willing and able to comply with scheduled visits, treatment plan, and other study procedures. Dyads using other hGH injection devices other than the current reusable Genotropin® Pen were excluded.

Written informed consent was obtained for subjects prior to screening. Upon screening and prior to introduction to the new disposable pen (Visit 1), parent–child dyads completed Section I of the IPAQ to assess their opinion/view regarding the ease of using their current reusable pen. Next, subjects and caregivers were instructed in the proper use of the new disposable pen and were asked to use the new pen for two months. A telephone follow-up contact (Visit 2) occurred at day 7 to assess safety and address any subject questions. After two months of using the new disposable pen, dyads were asked to return to the clinic to complete Sections I and II of the IPAQ (Visit 3) regarding their experience with the new disposable pen (i.e., ease of use and preference for a specific injection pen, respectively). Instructions on questionnaire completion were provided to all dyads by trained personnel.

### Clinical and patient-reported outcome measures

The IPAQ is a 29-item instrument that can be used to measure preference for an injection device from the perspective of the subject. The IPAQ is divided into two sections. Section I is designed to measure ease of use attributes (13 items) and “overall ease of use” (1 item) of a single pen. Items in Section I are rated on 5 point scale from “very easy” to “very difficult”. Section II compares the ease of use of two pens and contains the same ease of use attributes (13 items) and overall ease of use (1 item) as Section I. Response options include “Genotropin® Pen easier to use”, “New injection pen easier to use” or “no difference”. An additional item is included in Section II, which asks dyads to rate their overall preference for a device as “Prefer Genotropin® Pen”, “Prefer new injection pen” or “no preference”.

Demographic and clinical variables such as date of birth, gender, race, ethnicity, height, weight and primary diagnosis were collected at screening/baseline.

### Psychometric analysis

Analyses were performed to examine the following psychometric characteristics of the IPAQ in measuring ease of use/convenience and preference for injection pens: 1) item characteristics, 2) factor structure, 3) reliability, and 4) validity. Responsiveness analyses were not conducted because subjects did not evaluate the same injection pen using the IPAQ at two time points during this trial, which is required for this type of analysis. Analyses were conducted: 1) using data from dyads that completed the IPAQ for at least one time point and 2) separately for the individual injection pens. All data analyses were performed using SAS statistical software version 9.1 (Cary, NC).

#### Item characteristics

Characteristics of individual items of the IPAQ were examined by calculating the mean, minimum possible and maximum possible response (i.e., floor and ceiling effects), range, item frequency and item- to-item correlations. For Section I of the IPAQ, assessments for injection pens were conducted separately. Item analysis for Section II of the IPAQ examining item scores by injection pen preference was also conducted.

#### Factor structure and scoring

Confirmatory factor analysis (CFA) was used to examine the hypothesized structure of the IPAQ based on the conceptual framework developed through dyad interviews. Items within each of the four dimensions should be uni-dimensional. A CFA was conducted to fit a 4-factor model where each item was associated with one pre-defined factor. Overall model fit statistics were examined, as well as standardized regression coefficients (factor loadings) for each item. Good model-fit is indicated when Bentler Comparative Fit Index (CFI) is above 0.90 [13]. In addition, Root Mean Square Error of Approximation (RMSEA) should be below 0.05 as indication of good model-fit, or below 0.08 as acceptable model fit.

#### Subscale characteristics

To assess measurement properties of the resulting subscales from the factor analysis, subscale scores were calculated according to the proposed scoring guidelines, with higher scores indicating better outcomes. Distributional characteristics of the subscales were assessed, including means, floor and ceiling effects, and ranges. The subscale to subscale correlations also were assessed.

#### Reliability

Cronbach’s alpha was used to assess internal consistency of subscales generated by the CFA and total score for the IPAQ. Coefficient values that are greater than 0.70 are generally considered acceptable for aggregate data [14,15].

#### Validity

Spearman rank correlation coefficients were used to evaluate construct validity of the IPAQ through correlations between the IPAQ subscales and the “overall ease of use” item (Item 1 of Section I). Construct validity was supported when a specific subscale is substantially correlated (>0.40) with “overall ease of use” item. For interpretation, guidelines suggested by Cohen [16]were used, where absolute correlation values between 0.10 and 0.29 are considered weak, 0.30 to 0.49 are considered moderate, and 0.50 to 1.00 are considered strong.

Logistic regression analyses were performed to assess the relationship between IPAQ subscales and preference for a specific injection pen and to determine whether the predicting subscales (i.e., pen characteristics) are the same regardless of which injection pen dyads preferred. The first analysis evaluated the relationship between preference for an injection pen at Visit 3 and subscale scores for the reusable injection pen at Visit 1. Injection pen preference was categorized as “prefer reusable pen” and “prefer new disposable pen or no preference”. The regression model was: injection pen preference = score for preparing the pen + score for setting the dose + score for injecting the medicine + score for maintaining the pen + age + gender. The second analysis evaluated the relationship between preference for an injection pen at Visit 3 and subscale scores for the new disposable pen at Visit 3. Injection pen preference was categorized as “prefer new disposable pen” and “prefer reusable pen or no preference”. The regression model was: injection pen preference = score for preparing the pen + score for setting the dose + score for injecting the medicine + score for maintaining the pen + age + gender.

Tests of general association using the Mantel-Haenzel estimate of the common odds ratio also were used to assess the relationship between IPAQ comparative ease of use items and preference for a specific injection pen to determine if a rating for individual attributes is associated with preference for an injection pen. Injection pen preference was categorized as “prefer reusable pen or no preference” or “prefer new disposable pen” and ease of use items were categorized as “reusable pen easier to use or no difference” or “new disposable pen easier to use”. The Mantel-Haenzel tests were run for each individual injection pen attribute. The attributes are: attaching/removing needle, preparing the injection pen, mixing the medicine, removing the air bubbles, remembering the dose, setting the dose, changing the dose for prescription changes, using the needle guard, injecting the medicine, knowing when the injection was finished, handling the pen, storing the pen, disposing of the cartridge/pen and “overall ease of use”. Gender was included in the Mantel-Haenzel tests as a stratification factor.

#### Additional analyses

To determine which items most influence preference, between-group differences in item ratings were assessed using chi-square analyses, where groups were defined based on preferences for a device (i.e., prefer reusable pens, prefer new disposable pen). Visit 1 ratings were used for dyads who preferred reusable pen and were compared with Visit 3 ratings for those who preferred new disposable pen. Item responses were grouped into two categories: “very easy” and “not very easy”, which includes all other response options (“somewhat easy”, “neither easy nor difficult”, “somewhat difficult” and “very difficult”). In addition, differences in item ratings were assessed between Visits 1 and 3 within each preference groups (i.e., prefer reusable pen or prefer new disposable pen) using ICC analyses, to determine which items most impacted preference.

## Results

### Dyad characteristics

A total of 136 dyads were included in the psychometric analysis. This included one dyad that completed the assessments for Visit 3 outside of the specified time window. Characteristics of the child (subject) member of the dyad are summarized in Table 1. Subjects were primarily boys (66.9%), white (84.6%), non-Hispanic/Latino (91.2%) and were an average of 12.3 years old. Growth hormone deficiency (52.9%) was the most frequently reported primary diagnosis, followed by small for gestational age (20.6%), and growth retardation (11.0%). Subjects had been diagnosed for an average of 5.6 years (sd 4.3) prior to the study.

**Table 1:** Demographic and clinical characteristics of hGH recipient at screening/baseline

	**Total (N = 136)**
Age (years), mean (SD)	12.3 (2.4)
Gender, n (%male)	91 (66.9%)
Height (inches), mean (SD)	57.5 (5.6)
Weight (pounds), mean (SD)	92.9 (33.5)
Race, n (%)	
White	115 (84.6%)
Black	14 (10.3%)
Asian	2 (1.5%)
Other	5 (3.7%)
Ethnicity, n (%)	
Hispanic/Latino	12 (8.8%)
Not Hispanic/Latino	124 (91.2%)
Primary diagnosis, n (%)	
Dwarfism	1 (0.7%)
Fetal growth retardation	1 (0.7%)
Hypopituitarism	3 (2.2%)
Hypopituitarism fetal	1 (0.7%)
Small for gestational age	28 (20.6%)
Turner's syndrome	10 (7.4%)
Growth retardation	15 (11.0%)
Growth hormone deficiency	72 (52.9%)
Body height below normal	4 (2.9%)
Silver-Russell syndrome	1 (0.7%)
Time since diagnosis (years), mean (SD)	5.6 (4.3)

Almost all of the adult dyad members (82.4%) were subjects’ mothers. The majority of the adult dyad members were responsible for preparing the injection and administering the injection (82.4% and 73.5%, respectively) (Table 2). On average, dyads reported having used the reusable pen for 4.0 years (Table 2). The majority of subjects use the 12 mg Genotropin® reusable pen (94.1%).

**Table 2:** Injection pen and IPAQ respondent characteristics

	**Total (N = 136)**
Caregiver’s relationship to child, n (%)	
Mother	112 (82.4%)
Father	22 (16.2%)
Grandfather	1 (0.7%)
Stepfather	1 (0.7%)
Length of time using reusable pen(years), mean (SD)	4.0 (3.1)
Type of Pen, n (%)	
5 MG	8 (5.9%)
12 MG	128 (94.1%)
Person responsible for preparing shot, n (%)	
Child	24 (17.6%)
Mother	87 (64.0%)
Father	22 (16.2%)
Grandmother	1 (0.7%)
Stepfather	1 (0.7%)
Other	1 (0.7%)
Person responsible for giving the shot, n (%)	
Child	36 (26.5%)
Mother	77 (56.6%)
Father	21 (15.4%)
Grandmother	1 (0.7%)
Stepfather	1 (0.7%)

### Item characteristics

IPAQ item scores range from 1 (very difficult) to 5 (very easy). Items 2 g (“changing the dose when the doctor changes the prescription” and 2 h (“using the needle guard”) followed a skip pattern, where subjects who did not have a dose change or did not use the needle guard skipped these items. For the reusable and new disposable injection pens, 21.3% and 80.6%, respectively, did not report a change in dosage during the observation period. In addition, approximately 20% of subjects did not use a needle guard with either pen.

For the reusable pen, mean Section I IPAQ scores ranged from 3.4 to 4.8 (Table 3). For eight of the thirteen IPAQ items, greater than 50% of dyads scored at the ceiling (“very easy”). Mean Section I IPAQ scores for the new disposable pen ranged from 4.1 to 4.8 (Table 4). In the case of the new disposable pen, all 13 items had 50% of more respondents reporting at the ceiling (very easy).

**Table 3:** **IPAQ item distributional characteristics for Section I (reusable pen)**^**1,2**^

	**N**	**Mean**	**SD**	**Floor (%)**	**Ceiling (%)**	**Range**
1. Ease or difficulty of using the injection pen overall	136	4.2	0.9	1 (0.7%)	58 (42.6%)	1.0-5.0
2a. Attaching and removing the needle	136	4.6	0.8	0 (0.0%)	98 (72.1%)	2.0-5.0
2b. Preparing the injection pen by changing the cartridge or getting a new injection pen	136	3.8	1.1	2 (1.5%)	38 (27.9%)	1.0-5.0
2c. Mixing the medicine	136	4.2	1.0	0 (0.0%)	67 (49.3%)	2.0-5.0
2d. Removing the air bubbles	136	3.7	1.1	4 (2.9%)	42 (30.9%)	1.0-5.0
2e. Remembering the dose that was prescribed by the doctor	136	4.8	0.6	0 (0.0%)	112 (82.4%)	3.0-5.0
2f. Setting the dose	136	4.7	0.8	1 (0.7%)	108 (79.4%)	1.0-5.0
2g. Changing the dose when the doctor changes the prescription^3^	107	4.8	0.6	0 (0.0%)	90 (84.1%)	2.0-5.0
2h. Using the needle guard^3^	111	4.6	0.6	0 (0.0%)	78 (70.3%)	2.0-5.0
2i. Injecting the medicine	136	4.3	1.0	3 (2.2%)	71 (52.2%)	1.0-5.0
2j. Knowing when the injection pen has finished injecting the medicine	136	3.4	1.4	12 (8.8%)	42 (30.9%)	1.0-5.0
2k. Handling the injection pen while preparing and injecting the medicine	136	4.2	0.9	1 (0.7%)	62 (45.6%)	1.0-5.0
2l. Storing the injection pen in the refrigerator	136	4.7	0.8	0 (0.0%)	110 (80.9%)	2.0-5.0
2m. Disposing of the cartridge or disposing of the injection pen	136	4.6	0.7	0 (0.0%)	99 (72.8%)	2.0-5.0

^1^Assessment of reusable pen (Visit 1).

^2^Floor indicates “Very Difficult” and ceiling indicates “Very Easy”.

^3^Percentage based on the number of dyads who changed the dose (N = 107) or used the needle guard (N = 111).

**Table 4:** **IPAQ item distributional characteristics for Section I (new disposable pen)**^**1,2**^

	**N**	**Mean**	**SD**	**Floor (%)**	**Ceiling (%)**	**Range**
1. Ease or difficulty of using the injection pen overall	134	4.3	1.0	1 (0.7%)	75 (56.0%)	1.0-5.0
2a. Attaching and removing the needle	134	4.8	0.6	0 (0.0%)	116 (86.6%)	2.0-5.0
2b. Preparing the injection pen by changing the cartridge or getting a new injection pen	134	4.5	0.8	1 (0.7%)	91 (67.9%)	1.0-5.0
2c. Mixing the medicine	134	4.6	0.6	0 (0.0%)	95 (70.9%)	2.0-5.0
2d. Removing the air bubbles	134	4.6	0.7	0 (0.0%)	94 (70.1%)	2.0-5.0
2e. Remembering the dose that was prescribed by the doctor	134	4.8	0.6	1 (0.7%)	117 (87.3%)	1.0-5.0
2f. Setting the dose	134	4.6	0.8	1 (0.7%)	100 (74.6%)	1.0-5.0
2g. Changing the dose when the doctor changes the prescription	26	4.7	0.8	0 (0.0%)	22 (84.6%)	2.0-5.0
2h. Using the needle guard^3^	107	4.5	0.8	0 (0.0%)	76 (71.0%)	2.0-5.0
2i. Injecting the medicine	134	4.1	1.2	3 (2.2%)	69 (51.5%)	1.0-5.0
2j. Knowing when the injection pen has finished injecting the medicine	134	4.1	1.2	4 (3.0%)	71 (53.0%)	1.0-5.0
2k. Handling the injection pen while preparing and injecting the medicine	134	4.3	1.1	1 (0.7%)	82 (61.2%)	1.0-5.0
2l. Storing the injection pen in the refrigerator	134	4.4	1.1	3 (2.2%)	98 (73.1%)	1.0-5.0
2m. Disposing of the cartridge or disposing of the injection pen	133	4.5	0.9	1 (0.8%)	99 (74.4%)	1.0-5.0

^1^Assessment of new disposable pen (Visit 3).

^2^Floor indicates “Very Difficult” and ceiling indicates “Very Easy”.

^3^Percentage based on the number of dyads who changed the dose (N = 26) or used the needle guard (N = 107).

### Factor analysis and scoring algorithm

CFA was conducted to substantiate a second order model, based on the conceptual framework for the IPAQ. This model, based on qualitative data from the development of the IPAQ, shows individual items (injection pen attributes) fitting into 4 subscales (preparing the pen, setting the dose, injecting the medicine and maintaining the pen) and the 4 subscales fit under a total ease of use domain. Findings from the CFA suggest that Item 2 k (“Handing the injection pen while preparing and injecting the medicine”) belongs in the subscale for injecting the medicine, rather than the subscale for maintaining the injection pen, as in the conceptual model based on dyad interviews. As such, Item 2 k was included in the “injecting the medicine” subscale.

CFA of the IPAQ showed evidence of a second order factor model (Tables 5 and 6). For the first order factor model [preparing the pen (4 items), setting the dose (3 items), injecting the medicine (4 items) and maintaining the pen (2 items)], loadings for all items within each factor exceeded 0.40 with the majority being higher than 0.60. Loading ranged from 0.57 to 0.85 for the reusable pen (Table 5) and from 0.59 to 0.96 for the new disposable pen (Table 6). The subscales loaded on the total ease of use domain, with loadings ranging from 0.79 to 0.94 for the reusable pen (Table 5) and from 0.63 to 0.97 for the new disposable pen (Table 6). Fit indices for the reusable pen were: CFI = 0.952 and RMSEA = 0.080. Fit indices for the new disposable pen were: CFI = 0.984 and RMSEA = 0.051.

**Table 5:** Confirmatory factor analysis of IPAQ: factor loading for ease of use for reusable pen (N = 136)

**FIRST ORDER (IPAQ Items)**	**IPAQ Ease of Use Subscales (standardized coefficient)**				
	**Preparing the pen**	**Setting dose**	**Injecting the medicine**	**Maintaining the pen**	**Total IPAQ**
2a. Attaching and removing the needle	0.57	---	---	---	---
2b. Preparing the injection pen by changing the cartridge or getting a new injection pen	0.68	---	---	---	---
2c. Mixing the medicine	0.84	---	---	---	---
2d. Removing the air bubbles	0.79	---	---	---	---
2e. Remembering the dose that was prescribed by the doctor	---	0.61	---	---	---
2f. Setting the dose	---	0.62	---	---	---
2g. Changing the dose when the doctor changes the prescription	---	0.80	---	---	---
2h. Using the needle guard	---	---	0.74	---	---
2i. Injecting the medicine	---	---	0.71	---	---
2j. Knowing when the injection pen has finished injecting the medicine	---	---	0.73	---	---
2k. Handling the injection pen while preparing and injecting the medicine	---	---	0.76	---	---
2l. Storing the injection pen in the refrigerator	---	---	---	0.85	---
2m. Disposing of the cartridge or disposing of the injection pen	---	---	---	0.78	---
**SECOND ORDER (IPAQ Subscales)**	---	---	---	---	---
Preparing the pen	---	---	---	---	0.79
Setting the dose	---	---	---	---	0.94
Injecting the medicine	---	---	---	---	0.91
Maintaining the pen	---	---	---	---	0.93

Item 1 is a single item subscale and is not included in the analysis.

CFI = 0.984.

Root Mean Square Error of Approximation (RMSEA) = 0.051.

**Table 6:** Confirmatory factor analysis of IPAQ: factor loading for ease of use for new disposable pen (N = 134)

**FIRST ORDER (IPAQ Items)**	**IPAQ Ease of Use Scales (standardized coefficient)**				
	**Preparing the pen**	**Setting dose**	**Injecting the medicine**	**Maintaining the pen**	**Total IPAQ**
2a. Attaching and removing the needle	0.68	---	---	---	---
2b. Preparing the injection pen by changing the cartridge or getting a new injection pen	0.82	---	---	---	---
2c. Mixing the medicine	0.77	---	---	---	---
2d. Removing the air bubbles	0.72	---	---	---	---
2e. Remembering the dose that was prescribed by the doctor	---	0.79	---	---	---
2f. Setting the dose	---	0.77	---	---	---
2g. Changing the dose when the doctor changes the prescription	---	0.59	---	---	---
2h. Using the needle guard	---	---	0.88	---	---
2i. Injecting the medicine	---	---	0.88	---	---
2j. Knowing when the injection pen has finished injecting the medicine	---	---	0.70	---	---
2k. Handling the injection pen while preparing and injecting the medicine	---	---	0.80	---	---
2l. Storing the injection pen in the refrigerator	---	---	---	0.69	---
2m. Disposing of the cartridge or disposing of the injection pen	---	---	---	0.96	---
**SECOND ORDER (IPAQ Scales)**	---	---	---	---	---
Preparing the pen	---	---	---	---	0.87
Setting the dose	---	---	---	---	0.97
Injecting the medicine	---	---	---	---	0.86
Maintaining the pen	---	---	---	---	0.63

Item 1 is a single item subscale and is not included in the analysis.

CFI = 0.984.

Root Mean Square Error of Approximation (RMSEA) = 0.051.

The final scoring algorithm for the 13 Section I IPAQ items, excluding Item 1, therefore, consisted of four subscale scores (preparing the pen, setting the dose, injecting the medicine and maintaining the pen). Each subscale score is calculated by summing the value of individual items within a subscale. A total score (total IPAQ score), representing the average of the 4 subscales, also can be calculated. All subscale scores were transformed to a 0 to 100 scale, with higher scores reflecting greater ease of use.

### Subscale characteristics

Table 7 and b shows the distributional characteristics (i.e. mean, floor and ceiling effects, and range) of the IPAQ subscales for the reusable pen and the new disposable pen, respectively. Subscales are scored from 0 to100 with higher scores representing better outcomes (i.e., easier to use). For the reusable pen (Table 7), mean subscale scores ranged from 76.4 (preparing the pen) to 93.0 (setting the dose). For two of the subscales, greater than 60% of the dyads scored at the ceiling (setting the dose 62.5% and maintaining the pen 66.9%). For the new disposable pen (Table 8), mean subscale scores ranged from 80.6 (injecting the medicine) to 92.5 (setting the dose). For two of the subscales, greater than 60% of the dyads scored at the ceiling (maintaining the pen 64.9% and setting the dose 69.4%).

**Table 7:** **IPAQ scale distributional characteristics - assessment eeusable pen (Visit 1)**^**1**^

**Subscale/Total Score**	**N**	**Mean**	**SD**	**Floor (%)**	**Ceiling (%)**	**Range**
Overall ease of use	136	79.6	22.2	1 (0.7%)	58 (42.6%)	0.0-100.0
Preparing the pen	136	76.4	18.2	0 (0.0%)	21 (15.4%)	31.3-100.0
Setting the dose	136	93.0	12.4	0 (0.0%)	85 (62.5%)	33.3-100.0
Injecting the medicine	136	77.1	19.5	1 (0.7%)	25 (18.4%)	0.0-100.0
Maintaining the pen	136	91.1	16.0	0 (0.0%)	91 (66.9%)	37.5-100.0
Total IPAQ score	136	84.4	12.8	0 (0.0%)	13 (9.6%)	42.7-100.0

^1^Ceiling indicates “Very Easy” for all items in a subscale and floor indicates “Very Difficult” for all items in a subscale.

**Table 8:** **IPAQ scale distributional characteristics - assessment new disposable pen (Visit 3)**^**1**^

**Subscale/Total Score**	**N**	**Mean**	**SD**	**Floor (%)**	**Ceiling (%)**	**Range**
Overall ease of use	134	82.6	23.8	1 (0.7%)	75 (56.0%)	0.0-100.0
Preparing pen	134	90.9	12.6	0 (0.0%)	64 (47.8%)	37.5-100.0
Setting the dose	134	92.5	14.5	0 (0.0%)	93 (69.4%)	25.0-100.0
Injecting the medicine	134	80.6	21.5	0 (0.0%)	47 (35.1%)	25.0-100.0
Maintaining the pen	134	86.2	21.9	0 (0.0%)	87 (64.9%)	12.5-100.0
Total IPAQ score	134	87.5	13.2	0 (0.0%)	30 (22.4%)	37.5-100.0

^1^Ceiling indicates “Very Easy” for all items in a subscale and floor indicates “Very Difficult” for all items in a subscale.

Subscale to subscale correlations for the reusable pen, moderate to strong, significant correlations were observed between all the subscales (range 0.34 to 0.56, all p < 0.0001), and significant correlations were observed between the total IPAQ score and all subscales (range 0.63 – 0.86, all p < 0.0001). For the new disposable pen, moderate to strong, significant correlations were observed between most of the subscales (range 0.23 to 0.53, all p < 0.01), and moderate to strong, significant correlations were observed between the total IPAQ score and all subscales (range 0.60 – 0.84, all p < 0.0001).

### Reliability

Total IPAQ scores demonstrated high internal consistency (Cronbach’s alpha = 0.85) for both Visit 1 (Geontropin Pen assessment) and Visit 3 (new disposable pen assessment) (Table 9). Cronbach’s alpha for the subscales ranged from 0.58 to 0.73 (reusable pen assessment); with two scales (setting the dose and maintaining the pen) having alphas less than 0.70. For the new disposable pen assessment, Cronbach’s alpha for the subscales ranged from 0.53 to 0.82; with three scales having alphas less than 0.70 – likely due to the small number of items within each scale (2 or 3 items) and the large ceiling effects.

**Table 9:** IPAQ internal consistency reliability

	**Genotropin® Pen**^**1**^		**New Disposable Pen**^**2**^	
	**# of Items**	**Cronbach’s Alpha**	**# of Items**	**Cronbach’s Alpha**
Overall ease of use	1	---	1	---
Preparing the pen	4	0.70	4	0.68
Setting the dose	3	0.58	3	0.53
Injecting the medicine	4	0.73	4	0.82
Maintaining the pen	2	0.65	2	0.65
Total IPAQ score	13	0.85	13	0.85

^**1**^ N = 136, Visit 1.

^**2**^ N = 134, Visit 3.

### Validity

#### Construct validity

For the reusable pen, moderate to strong, significant correlations were found with three of the IPAQ subscales and the “overall ease of use” item (preparing the pen, setting the dose and injecting the medicine) (range 0.49 - 0.52, p < 0.0001). Strong, significant correlations also were seen between these three subscales and the “overall ease of use” item for the new disposable pen (range 0.53 to 0.65, p < 0.0001). For both the reusable and new disposable pens, total IPAQ scores correlated strongly with the “overall ease of use” item (0.59 and 0.67, respectively; both p < 0.0001).

#### Predictive validity

##### Preference for the reusable Pen: IPAQ scales

In the pre-specified logistic regression model [i.e., preference for the reusable pen vs. (preference for the disposable pen + no preference) = age + gender + 4 IPAQ subscales], results indicate that none of the individual subscales predict preference for the reusable pen. Being male, however, predicted preference – with males being less likely to prefer the reusable pen (0.409 point estimate; confidence interval 0.180 to 0.927). Similar findings were seen for the post-hoc logistic regression model, which was the same as the first with the inclusion of the “overall ease of use” item. Findings from another post-hoc model [i.e., preference for the reusable pen vs. (preference for the disposable pen + no preference) = age + gender + total IPAQ score] indicate that neither total IPAQ score, age nor gender predict preference for the reusable pen.

##### Preference for the New disposable Pen: IPAQ scales

In the pre-specified logistic regression model [i.e., preference for the new disposable pen vs. (preference for the reusable pen + no preference) = age + gender + 4 IPAQ subscales], results indicate that none of the individual subscales predict preference for the new disposable pen. When including the “overall ease of use” item (Item 1) in the pre-specified model, “overall ease of use” predicted preference for the new disposable pen. Results indicate that for each one-unit increase in “overall ease of use” item score, we expect to see a 6.3% (1.063 point estimate; confidence interval 1.028 to 1.099) increase in the odds of preferring the new disposable pen. When including only the total IPAQ score in the model, the total score predicted preference for the new disposable pen. Results indicate that for each one-unit increase in total IPAQ score, we expect to see a 10.6% (1.106 point estimate; confidence interval 1.062 to 1.153) increase in the odds of preferring the new disposable pen.

##### Mantel-haenzel estimate of common odds ratios: IPAQ items

Analysis was conducted to assess the relationship between ratings of injection pens as “easier to use” and preference. Three ways of categorizing preference were evaluated: 1) preference for the reusable pen vs. (preference for the disposable pen + no preference), 2) preference for the new disposable pen vs. (preference for the reusable pen + no preference) and 3) preference for the reusable pen vs. preference for the disposable pen. In general, similar findings were seen among the models, where rating the new disposable pen as “easier to use” overall being the strongest predictor of preference for the new disposable pen (odds ratio for Model 1: 46.4). The individual attributes that were rated as “easier to use” for the new disposable pen and that were the strongest predictors for preference for the new disposable pen were: setting the dose, handling the pen, removing the air bubbles and injecting the medicine (odds ratios for Model 1: 25.0, 18.9, 17.8 and 17.7, respectively).

### Additional analyses

Comparing dyads who preferred the reusable pen to those who preferred the new disposable pen, a higher percentage of dyads rated 8 of the 13 attributes “very easy” for the new disposable pen compared to “very easy” for the reusable pen (attaching and removing the needle, preparing the injection pen by changing the cartridge or getting a new injection pen, mixing the medicine, removing the air bubbles, remembering the dose prescribed by the doctor, knowing when the injection pen has finished injecting the medicine, handling the injection pen while preparing and injecting the medicine; all <0.05). Percentages of dyads who preferred the new disposable pen (n = 79) and rated the new disposable pen “very easy” to use ranged from 62.0% to 94.9% compared to 30.4% to 87.0% for those who preferred the reusable pen (n = 46) and rated the reusable pen “very easy” to use. For Items 2b and 2d (preparing the injection pen and removing the air bubbles) it is interesting to note that of dyads who preferred the reusable pen, only 30% rated these attributes as “very easy”. This may suggest that for some attributes, habituation may influence preference (i.e., it may not be easy, but the person is used to it and prefers to keep using the pen).

In this analysis, an ICC of less than 0.30 was considered the cut-off for showing a relationship to preference (i.e., correlations <0.30 suggest a relationship to preference) by demonstrating inconsistency in responses across both injection pens (e.g., having higher ratings for the “remembering the dose” item when assessing the reusable pen compared to the new disposable pen for dyads who preferred the reusable pen). For those that preferred the reusable pen, all but two attributes (ease or difficulty of using the injection pen overall and attaching and removing the needle) were related to preference based on the ICCs (Tables 10). Six attributes demonstrated significant differences in scores between the two injection pens (mixing the medicine, remembering the dose prescribed by the doctor, changing the dose when the doctor changes the prescription, using the needle guard, knowing when the injection pen has finished injecting the medicine, and handling the injection pen while preparing and injecting the medicine). For those that preferred the new disposable pen, all attributes were related to preference based on the ICCs (Table 11). All but 4 attributes (setting the dose, changing the dose when the doctor changes the prescription, handling the injection pen while preparing and injecting the medicine, and storing the injection pen in the refrigerator) demonstrated significant differences in scores between the two injection pens.

**Table 10:** ICCs of IPAQ items for dyads who preferred the Genotropin® Pen

**Item**	**N**	**Mean(SD) Visit 1**	**Mean(SD) Visit 3**	**Difference (SD)**^**1**^	**P value**	**ICC**
1. Ease or difficulty of using the injection pen overall	46	4.54 (0.84)	4.59 (0.88)	0.04 (1.01)	0.7717	0.31
2a. Attaching and removing the needle	46	3.80 (1.09)	4.07 (1.04)	0.26 (1.25)	0.1655	0.30
2b. Preparing the injection pen by changing the cartridge or getting a new injection pen	46	4.26 (1.00)	4.50 (0.69)	0.24 (1.02)	0.1171	0.29
2c. Mixing the medicine	46	3.74 (1.10)	4.33 (0.90)	0.59 (1.24)	0.0024	0.21
2d. Removing the air bubbles	46	4.72 (0.54)	4.65 (0.67)	−0.07 (0.80)	0.5831	0.15
2e. Remembering the dose that was prescribed by the doctor	46	4.80 (0.58)	4.17 (1.16)	−0.63 (1.32)	0.0023	−0.03
2f. Setting the dose	9	4.89 (0.33)	4.67 (0.71)	−0.22 (0.83)	0.4468	−0.14
2g. Changing the dose when the doctor changes the prescription	35	4.77 (0.55)	4.17 (1.15)	−0.60 (1.17)	0.0045	0.13
2h. Using the needle guard	46	4.30 (0.96)	3.28 (1.29)	−1.02 (1.63)	0.0001	−0.01
2i. Injecting the medicine	46	3.65 (1.29)	3.59 (1.36)	−0.07 (1.72)	0.7980	0.16
2j. Knowing when the injection pen has finished injecting the medicine	46	4.35 (0.90)	3.61 (1.27)	−0.74 (1.37)	0.0007	0.19
2k. Handling the injection pen while preparing and injecting the medicine	46	4.70 (0.76)	4.00 (1.41)	−0.70 (1.55)	0.0038	0.06
2l. Storing the injection pen in the refrigerator	46	4.61 (0.80)	4.41 (0.88)	−0.20 (1.09)	0.2288	0.17

^1^Difference = Visit 3 - Visit 1.

**Table 11:** ICCs of IPAQ items for dyads who preferred the New Disposable Pen

**Item**	**N**	**Mean(SD) Visit 1**	**Mean(SD) Visit 3**	**Difference (SD)**^**1**^	**P value**	**ICC**
1. Ease or difficulty of using the injection pen overall	79	4.59 (0.81)	4.92 (0.27)	0.33 (0.78)	0.0003	0.14
2a. Attaching and removing the needle	79	3.77 (1.01)	4.78 (0.44)	1.01 (1.06)	<0.0001	0.05
2b. Preparing the injection pen by changing the cartridge or getting a new injection pen	79	4.15 (0.93)	4.76 (0.51)	0.61 (1.01)	<0.0001	0.08
2c. Mixing the medicine	79	3.73 (1.16)	4.75 (0.54)	1.01 (1.14)	<0.0001	0.13
2d. Removing the air bubbles	79	4.76 (0.60)	4.95 (0.22)	0.19 (0.66)	0.0127	-.06
2e. Remembering the dose that was prescribed by the doctor	79	4.56 (0.92)	4.84 (0.46)	0.28 (0.96)	0.0118	0.12
2f. Setting the dose	13	4.54 (0.97)	4.85 (0.55)	0.31 (1.18)	0.3665	-.13
2g. Changing the dose when the doctor changes the prescription	61	4.56 (0.70)	4.75 (0.57)	0.20 (0.81)	0.0636	0.17
2h. Using the needle guard	79	4.23 (1.02)	4.54 (0.81)	0.32 (1.28)	0.0305	0.05
2i. Injecting the medicine	79	3.25 (1.36)	4.37 (0.92)	1.11 (1.46)	<0.0001	0.15
2j. Knowing when the injection pen has finished injecting the medicine	79	4.09 (0.94)	4.66 (0.71)	0.57 (1.12)	<0.0001	0.08
2k. Handling the injection pen while preparing and injecting the medicine	79	4.70 (0.72)	4.61 (0.87)	−0.09 (1.08)	0.4666	0.09
2l. Storing the injection pen in the refrigerator	78	4.59 (0.69)	4.60 (0.86)	0.01 (0.96)	0.9064	0.24

^1^Difference = Visit 3 - Visit 1.

## Summary

The IPAQ was specifically designed to measure ease of use and preference of Genotropin injection pens in dyads being treated with hGH. Previous qualitative work to develop the IPAQ in dyads using the reusable pen suggests that the IPAQ has 4 subscales measuring ease of use: preparing the pen, setting the dose, injecting the medicine and maintaining the pen. In addition, these subscales can be combined into a total score.

Individual responses for the ease of use items (Section I) and subscale scores demonstrated ceiling effects. These results may reflect the ease of these injection pens and the positive attitudes towards using these pens or prior experience taking hGH among the study sample. These findings are not unexpected and are consistent with other studies evaluating ease of use with injection devices where subject ratings are skewed towards the most favorable responses [3,17-21].

The CFA provide evidence supporting a second order factor solution for four subscales (preparing the pen, setting the dose, injecting the medicine and maintaining the pen) and a total score (total IPAQ score). These factor analysis results support the conceptual framework developed from previous qualitative research in patient dyads using the reusable pen [22].

The IPAQ did not demonstrate internal consistency reliability above the 0.70 threshold for group level comparisons for all subscales and this may have been due to the significant ceiling effects observed in the items. The findings indicated that the subscale and total IPAQ scores are internally consistent for only 2 and 1 (of 4 subscales) for the current reusable and new disposable pens, respectively (Cronbach’s alpha range 0.53 to 0.82). Cronbach’s alphas for the total IPAQ score for both pens are 0.85, exceeding acceptable levels of reliability for group comparisons. This study provides evidence supporting construct validity of the IPAQ in subjects being treated with hGH, where subscale and total IPAQ scores were moderately or strongly, significantly related to “overall ease of use” item assessments for each injection pen. In addition, evidence is provided for the predictive validity of the total IPAQ score predicting preference for the new disposable pen.

The IPAQ was developed for application in trials comparing ease of use and preference between these specific injection pens with regard to injection pen attributes. Findings from this research suggest the suitability for use of a total IPAQ score for ease of use and the single ease of use item. CFA supports the use of subscale scores to assess ease of use. The psychometric properties of these subscales, mainly the less than desirable internal consistency reliability of some of the subscales and the predictive validity findings, do not support the use of subscale scores alone. The subscale scores, however, do provide additional information regarding the performance of the pens as seen by the subject dyads. As such, it is recommended that the total IPAQ score is used as the primary ease of use endpoint because of its evidence on reliability and validity, and because it is a construct that is important to patients and their caregivers [22]. The subscale scores can be used to examine reasons for any observed differences in total IPAQ scores, and to provide more insight into injection pen attribute preferences. The IPAQ ease of use item may be useful in trials to get information about ease of use with different injection devices - though such application would require additional validation. Similarly, the single item ratings of overall ease of use can be used in clinical practice to get information on patient and caregiver impressions of ease of use for injection devices; and findings from this study suggest that the overall impression of ease of use is predictive of preference for the new disposable pen. The single-item measure is not recommended as a primary endpoint for clinical trials, however, since it is less reliable than the multi-item total IPAQ score.

In conclusion, the total IPAQ score for ease of use demonstrated good internal consistency reliability and good construct validity in measuring ease of use with the Genotropin® injection pens to administer hGH. Evidence of the psychometric properties of the IPAQ is important to support claims of potential treatment benefit for the FDA [11,23,24]. Since this study was conducted only in the USA and only in experienced Genotropin® Pen users, further research is needed to confirm these findings with Genotropin® and new disposable pens in other countries and in treatment naïve patients. Since hGH is also used in adults, a study of ease of use in that population would be of interest with regard to the IPAQs operating characteristics. Overall, the IPAQ is a useful measure for evaluating ease of use and preference for Genotropin® injection pens in clinical trials among patient dyads receiving hGH.

## Competing interests

Andreas Pleil, Natasa Rajicic and Judith Hey-Hadavi are employees of Pfizer Inc. Miriam Kimel and Julie McCormack were paid consultants to Pfizer in connection with the development of this manuscript and were employed by United BioSource Corporation at the time this research was conducted. The study was funded by Pfizer, Inc., New York, NY.

## Author’ contributions

AP, NR and JHH conceptualized the project and oversaw and provided scientific input regarding the development of the IPAQ. These three authors also designed and oversaw the conduct of the trial used for assessing the psychometric properties of the IPAQ. They also reviewed the statistical analysis plan (SAP) and psychometric data and provided scientific input. MK and JM designed and conducted the qualitative research and analyzed the qualitative data for developing the IPAQ as well as developed the SAP and analyzed the data for assessing the psychometric properties of the IPAQ. AP, MK, and JM also drafted the manuscript. All authors read and approved the final manuscript.
